# BAC Transgenic Mice Reveal Distal Cis-Regulatory Elements Governing BDNF Gene Expression

**DOI:** 10.1002/dvg.20606

**Published:** 2010-02-22

**Authors:** Indrek Koppel, Tamara Aid-Pavlidis, Kaur Jaanson, Mari Sepp, Kaia Palm, Tõnis Timmusk

**Affiliations:** Department of Gene Technology, Tallinn University of TechnologyTallinn, Estonia

**Keywords:** neurotrophin, transcription, promoter, BAC, transgenic mouse, kainic acid

## Abstract

Brain-derived neurotrophic factor (BDNF), a member of the neurotrophin family of neurotrophic factors, has important functions in the peripheral and central nervous system of vertebrates. We have generated bacterial artificial chromosome (BAC) transgenic mice harboring 207 kb of the rat *BDNF* (*rBDNF*) locus containing the gene, 13 kb of genomic sequences upstream of *BDNF* exon I, and 144 kb downstream of protein encoding exon IX, in which protein coding region was replaced with the *lacZ* reporter gene. This *BDNF*-BAC drove transgene expression in the brain, heart, and lung, recapitulating endogenous *BDNF* expression to a larger extent than shorter rat *BDNF* transgenes employed previously. Moreover, kainic acid induced the expression of the transgenic *BDNF* mRNA in the cerebral cortex and hippocampus through preferential activation of promoters I and IV, thus recapitulating neuronal activity-dependent transcription of the endogenous *BDNF* gene. genesis 48:214–219, 2010. © 2010 Wiley-Liss, Inc.

Brain-derived neurotrophic factor (BDNF), a member of the neurotrophin family of proteins, supports the survival and differentiation of certain neuronal populations during development (Bibel and Barde, 2000; Binder and Scharfman, 2004). In the adult, BDNF regulates long-term potentiation of synapses, thus playing a key role in long-term memory formation (Lu *et al.*, 2008). BDNF was originally isolated from the brain, but it is also expressed in the peripheral nervous system and non-neural tissues (Binder and Scharfman, 2004). Changes in *BDNF* gene expression accompany and contribute to the development of various disorders of the nervous system (Bibel and Barde, 2000).

The *BDNF* gene contains multiple promoters that initiate the transcription of a number of distinct mRNAs, each of which contains an alternative 5′ untranslated exon spliced to a common 3′ protein coding exon. In addition, the protein coding exon employs two different polyadenylation sites that give rise to mRNA species with 3′ untranslated regions (UTRs) of different lengths. Alternative promoter usage, differential splicing, and the use of two different polyadenylation sites within each of the transcription units generate at least 22 different *BDNF* mRNAs in rodents and 34 *BDNF* mRNAs in human that encode the same mature BDNF protein (Aid *et al.*, 2007; Pruunsild *et al.*, 2007). It has been shown that the subcellular localization of *BDNF* mRNAs and its regulation by neuronal activity depends on the 5′ exon and 3′ UTRs used in the transcript (An *et al.*, 2008; Chiaruttini *et al.*, 2008). In addition, it has been shown that *BDNF* mRNAs containing the short 3′ UTRs are more enriched in polysomal fraction isolated from total brain than *BDNF* mRNAs with the long 3′ UTRs suggesting that they are more efficiently translated (Timmusk *et al.*, 1994). Numerous regulatory elements involved in the regulation of *BDNF* expression in vitro and in vivo have been identified and characterized in different *BDNF* promoters. Transcription factors such as REST (Timmusk *et al.*, 1999; Zuccato *et al.*, 2003), CREB (Shieh *et al.*, 1998; Tao *et al.*, 1998), NFkB (Lipskyet *al.*, 2001), MEF2 (Flavell *et al.*, 2008), NPAS4 (Lin *et al.*, 2008), bHLHB2 (Jiang *et al.*, 2008), and MeCP2 (Chen *et al.*, 2003; Marti-nowich *et al.*, 2003) have been shown to regulate *BDNF* expression in a promoter-specific manner. However, the genomic regions including all necessary *cis*-acting elements responsible for the tissue-specific and activity-dependent *BDNF* gene regulation in vivo remain poorly characterized. A few studies have addressed these issues using transgenic mouse models (Funakoshi *et al.*, 1998; Guillemot *et al.*, 2007; Koppel *et al.*, 2009; Timmusk *etal.*, 1995, 1999).

In the present study, we have generated a transgenic mouse line using a bacterial artificial chromosome (BAC) clone containing 207 kb of rat *BDNF (rBDNF)* locus, encompassing the genomic region from 13 kb upstream of *rBDNF* exon I to 144 kb downstream of *rBDNF* coding exon. Neighboring genes of the *rBDNF* gene lie 151 kb upstream *(Ifna4)* and 190 kb downstream *(Sqrdl)* from it and therefore no additional genes/promoters were included in the BAC construct. To facilitate detection of transgene expression, we replaced the protein coding region of exon IX in the *rBDNF*-BAC with *lacZ* reporter gene (Fig. 1a). This should lead to the expression of functional β-galactosidase protein but not a BDNF-lacZ fusion protein. Functional β-galactosidase protein encoded by the *lacZ* reporter gene in *rBDNF-lacZ*-BAC was detected by transient expression in COS-7 cells (data not shown).

**FIG. 1:**
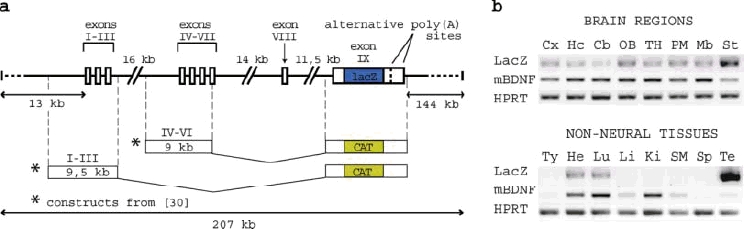
**(a)** Schematic diagram of the BAC construct used for generating *rBDNF-lacZ*-BAC transgenic mice (thick lines). White boxes represent untranslated sequences and the blue filled box represents *lacZ* reporter gene that replaces the *BDNF* coding sequence. *rBDNF-CAT* constructs (I–III and IV–VI) used by Timmusk *et al.* (1995) to generate *rBDNF* transgenic mice are shown with asterisks. **(b)** RT-PCR analysis of *rBDNF-lacZ* mRNA expression driven by *rBDNF* promoters in transgenic mouse tissues. Abbreviations: *mBDNF*, mouse *BDNF; HPRT*, hypoxanthine phosphoribosyltransferase 1; Cx, cortex; Hc, hippocampus; Cb, cerebellum; OB, olfactory bulb; TH, thalamus and hypothala-mus; PM, pons/medulla; Mb, midbrain; St, striatum; Ty, thymus; He, heart; Lu, lung; Li, liver; Ki, kidney; SM, skeletal muscle; Sp, spleen; Te, testis. [Color figure can be viewed in the online issue, which is available at http://www.interscience.wiley.com.]

In the *rBDNF-lacZ*-BAC transgenic line, the expression of *rBDNF-lacZ* mRNA was detected by RT-PCR in several brain regions and peripheral organs expressing endogenous mouse *BDNF (mBDNF)* mRNA (Fig. 1b). Specifically the expression of *rBDNF-lacZ* mRNA was detected in the brain regions of cortex, hippocampus, cerebellum, olfactory bulb, thalamus/hypothalamus, pons/medulla, midbrain, striatum, and also in the heart and lung. *rBDNF-lacZ* mRNA expression levels were not detected by RT-PCR in the thymus, liver, kidney, spleen, and skeletal muscle. Particularly high expression of the transgene was observed in the testis.

In the adult brain of the *rBDNF-lacZ*-BAC transgenic mice, in situ hybridization analysis revealed intense labeling of both *rBDNF-lacZ* and endogenous *mBDNF* mRNAs in the cerebral cortex (Figs. 2a–f and 3g,h), olfactory nucleus (Fig. 2ab,), hippocampus (Figs. 2e,f and 3a–f), amygdala (Fig. 2e–f), nucleus of the lateral olfactory tract (Fig. 2i,j), and hypothalamic nuclei (Fig. 2e,f, and 2k–n) including mamillary nuclei (Fig. 2k,l). In the granular cell layer of the olfactory bulb (Fig. 2a,b), caudate putamen, and nucleus accumbens (Fig. 2c,d), high levels of *rBDNF-lacZ* mRNA were detected, whereas labeling of the endogenous *mBDNF* mRNA was indistinguishable from background signal. In the claustrum (Fig. 2c,d) and hypothalamus (Fig. 2e,f), *rBDNF-lacZ* mRNA expression levels were relatively lower than *mBDNF* mRNA levels. In the hippocampus, intensive *rBDNF-lacZ* labeling over scattered neurons in the CA1 and CA3 subfields (Fig. 3a,c) mirrored the expression of the endogenous *mBDNF* (Fig. 3b,d). However, in the granule cells of dentate gyrus that showed high expression of *mBDNF* mRNA (Figs. 2f and 3f) no expression of *rBDNF-lacZ* was detected (Figs. 2e and 3e). In the cortex, *rBDNF-lacZ* expression was observed in cingulate and somatosensory areas in layers II–III and V–VI (Figs. 2c,e and 3g), whereas endogenous *mBDNF* was expressed throughout layers II–VI (Figs. 2d,f and 3h). Expression of *rBDNF-lacZ* (Fig. 2g,o) and *mBDNF* (Fig. 2h,p) mRNA was detected also in cardiac blood vessels but not in ventricular myocardium (Fig. 2g,h). In lung tissue, the levels of both *rBDNF-lacZ* and *mBDNF* mRNA were below detection limits of our in situ hybridization analysis (data not shown).

**FIG. 2:**
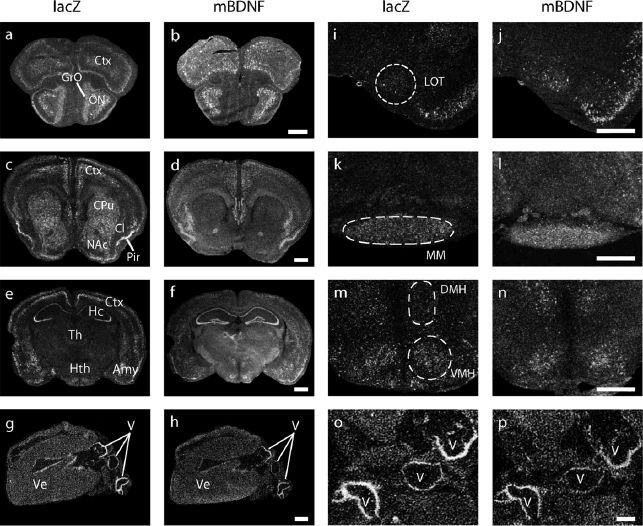
In situ hybridization analysis of *rBDNF-lacZ* mRNA expression in adult *rBDNF-lacZ*-BAC transgenic mouse brain and heart. Photomicrographs of 16 lm coronal brain (**a–f; i–n**) and transverse heart sections (**g,h,o,p**) hybridized with ^35^S-labeled *lacZ* or mouse endogenous *BDNF (mBDNF)* cRNA. The brain sections shown are at the levels of olfactory bulb (**a,b**), striatum (**c,d**), and hippocampus (**e,f**). (**i–n**) Magnifications of selected brain regions: LOT, nucleus of the lateral olfactory tract; MM, medial mammillary nucleus; DMH, dorsomedial hypothala-mic nucleus; VMH, ventromedial hypothalamic nucleus. (**o,p**) Magnifications of cardiac blood vessels. Scale bars: 1 mm (**a-h**) and 0.5 mm (**i–p**). Abbreviations: Ctx, cortex; GrO, olfactory bulb, granular cell layer; ON, olfactory nuclei; CPu, caudate putamen; Cl, claustrum; NAc, nucleus accumbens; Pir, piriform cortex; Hc, hippocampus; Th, thalamus; Hth, hypothalamus; Amy, amygdala; Ve, ventricle; V, cardiac blood vessel.

**FIG. 3:**
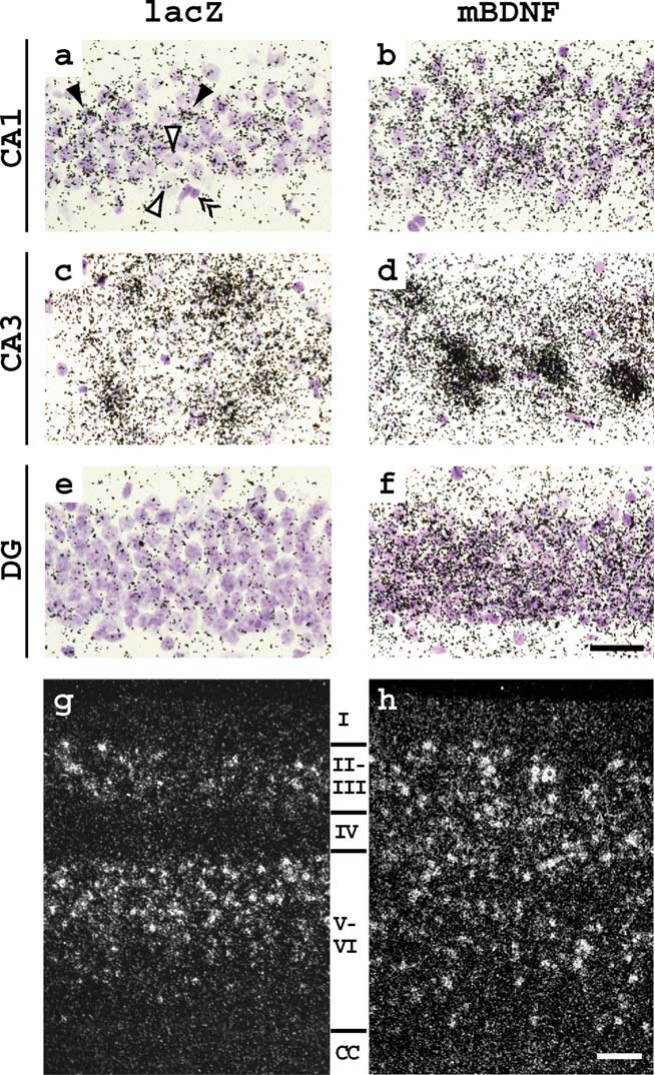
Cellular expression of *rBDNF-lacZ* mRNA in adult transgenic mouse brain: in situ hybridization analysis. (**a–f**) Bright-field photomicrographs of hippocampal subfields CA1, CA3, and dentate gyrus (DG). Hybridization probes are indicated above the columns; closed arrowheads indicate neurons with strong labeling; open arrowheads indicate neurons with weak or absent labeling; double arrowheads indicate a glial cell showing no labeling. (**g,h**) Distribution of *lacZ* and mouse *BDNF* labeling in cortical layers I–VI. Abbreviation: CC, corpus callosum. Scale bars: 20 μm (**a–f**) and 100 μm (**g,h**).

We also analyzed the expression and enzymatic activity of β-galactosidase protein in *rBDNF-lacZ*-BAC mouse tissues. Reporter activity was not detected in the brain or testis of the analyzed *rBDNF-lacZ*-BAC mouse line using X-gal staining assay. In addition, no expression of β-galactosidase protein was detected in the hippocampus, cortex, and testis of the transgenic animals using Western blot analysis (data not shown). These results suggest that β-galactosidase protein was either not translated from BAC-driven *rBDNF-lacZ* mRNAs or the levels of expression of the reporter protein remained below detection limits of the methods used in this study.

Kainic acid has been shown to induce *BDNF* mRNA expression in the adult rodent hippocampus and cerebral cortex (Zafra *et al.*, 1990) in a promoter-specific manner (Aid *et al.*, 2007; Timmusk *et al.*, 1993). Three hours after systemic injection of kainic acid, the levels of transgenic *rBDNF-lacZ* mRNA were increased in *rBDNF-lacZ*-BAC mice similarly to endogenous *mBDNF* mRNA (see Fig. 4). The elevated levels of *rBDNF-lacZ* and *mBDNF* mRNA expression were observed in cortical layers II–III and V–VI, hippocampal subfields CA1 and CA3, and in the amygdala. However, in contrast to endogenous *mBDNF*, induction of *rBDNF-lacZ* mRNA expression in the granule cells of the dentate gyrus was not observed (Fig. 4e,f). Quantitative real-time PCR analysis showed that induction pattern of different *rBDNF-lacZ* transcripts by kainic acid largely followed that of the endogenous *BDNF*: both transgenic and endogenous exon I and exon IV mRNAs transcribed from promoters I and IV, respectively, showed higher levels of induction than exon VI mRNAs transcribed from promoter VI (Fig. 4g,h). Similarly to untreated mice, β-galactosidase activity and protein expression was not detected in the cortex, hippocampus, and testis of kainate-treated *rBDNF-lacZ*-BAC mice (data not shown).

**FIG. 4:**
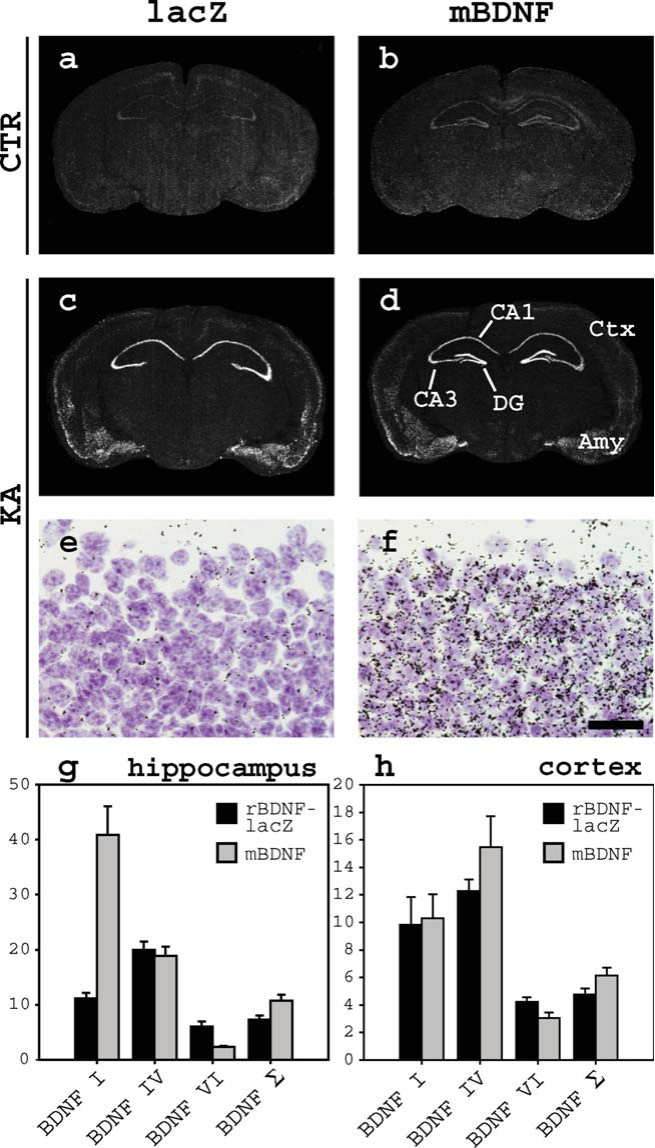
Induction of *rBDNF-lacZ* mRNA in transgenic mouse brain by kainic acid treatment. (**a–f**) In situ hybridization analysis with probes for transgenic *rBDNF-lacZ* and mouse endogenous *(mBDNF)* mRNA. Autoradiographs of sections from vehicle-treated (**a,b**) and kainate-treated animals (**c–f**) are shown. Dark-field autoradiographs of coronal sections (**a–d**); high magnification bright-field photomicrographs of the dentate gyrus (**e,f**). Scale bar: 20 μm (**e,f**). (**g,h**) Quantitative real-time PCR analysis of *rBDNF-lacZ* and endogenous *mBDNF* mRNA expression in the hippocampus (**g**) and cerebral cortex (**h**) of transgenic mice, expressed as fold difference relative to mRNA levels in vehicle-treated mice. Shown are transcripts containing exons I, IV, VI, and total *BDNF* mRNA (BDNF Σ). Error bars represent standard deviation of three RT-PCR experiments. Abbreviations: CTR, vehicle-treated control mice; KA, kainate-treated mice; CA1, CA3, hippocampal subfields; DG, dentate gyrus; Ctx, cortex; Amy, amygdala.

Transgenic mice expressing reporter genes under the control of various regulatory regions of the *rBDNF* gene have been described previously. *rBDNF-CAT* transgenic mice carrying 9 kb of genomic sequence comprising one or more *BDNF* 5′ untranslated exons were reported in (Timmusk *et al.*, 1995). These transgenic mice (Fig. 1a) recapitulated *BDNF* expression in most brain regions and in the thymus. However, *BDNF* IV–VI construct failed to recapitulate *BDNF* expression in the cerebellum, heart, and other peripheral tissues (Timmusk *et al.*, 1995) where *BDNF* transcripts IV and VI are endogenously expressed (Aid *et al.*, 2007; Pruunsild *et al.*, 2007; Timmusk *et al.*, 1993). Here we demonstrate that *rBDNF-lacZ*-BAC including 50 kb of the *rBDNF* gene, 13 kb of upstream and 144 kb of downstream sequences are not sufficient to drive *EGFP* (enhanced green fluorescent protein) reporter gene expression in the heart (Koppel *et al.*, 2009). Expression of *rBDNF-lacZ* mRNA in the heart of *rBDNF-lacZ*-BAC transgenic mice reported here (with 144 kb region 3′ of the *r*BDNF contains regulatory elements necessary for recapitulation of endogenous *BDNF* expression in the brain, heart, and lung, indicating that regulatory elements governing *BDNF* mRNA expression in these tissues are located within the 207 kb rat genomic sequence of the transgene. In addition, neuronal activity induced expression of *rBDNF-lacZ* mRNA in a promotor-specific manner in the *rBDNF-lacZ*-BAC mice, mimicking induction of the respective 5′ exon-specific transcripts of endogenous *BDNF*.

Recently, we have shown that human *BDNF-EGFP*-BAC covering 67 kb of the human *BDNF (hBDNF)* gene, 84 kb of upstream and 17 kb of downstream sequences gene) suggests that a heart-specific regulatory element is located within 18-144 kb 3′ of *BDNF* gene. However, this prediction should be treated with caution as regulatory regions of *BDNF* genes of different species are compared. On the other hand, neither *hBDNF-EGFP-BAC* (Koppel *et al.*, 2009) nor *rBDNF-lacZ*-BAC could direct transgene expression to hippocampal dentate granule cells suggesting that the respective regulatory regions are located in genomic regions further than 84 kb upstream of *BDNF* exon I and 144 kb downstream of *BDNF* coding exon. Existence of remote cis-acting elements controlling *BDNF* transcription has been demonstrated by recent studies describing a regulatory region 850 kb upstream of human and mouse *BDNF* genes, disruption of which causes obesity, cognitive impairment, and hyperactivity (Gray *et al.*, 2006; Sha *et al.*, 2007).

In conclusion, we have generated transgenic mice containing *rBDNF-lacZ*-BAC transgene that recapitulated the expression of endogenous *BDNF* mRNA in the brain and peripheral tissues and neuronal activity-dependent regulation of *BDNF* mRNA in the adult cerebral cortex and hippocampus. This mouse model represents a useful tool for further mapping of proximal and distal regulatory elements in rodent *BDNF* gene in vivo.

## METHODS

*rBDNF-lacZ*-BAC transgenic mice were generated using BAC clone CH230-106M15 (Chori BACPAC Resources, Oakland, CA) modified to replace *rBDNF* coding sequence with the *lacZ* reporter gene (Red®/ET® homologous recombination technology, Gene Bridges, Heidelberg, Germany) (Muyrers *et al.*, 1999). The BAC clone contains 207 kb of the *rBDNF* genomic locus (GenBank: AC108236) including 50 kb of *rBDNF* gene, 13 kb of 5′ and 144 kb of 3′ flanking sequences (Fig. 1a). Purified *rBDNF-lacZ*-BAC was transfected into COS-7 cells by DEAE-dextran and tested for reporter activity using β-galactosidase assay. Transgenic mice were generated at the Karolinska Center for Transgene Technologies (Stockholm, Sweden) by injection of NotI-linearized *rBDNF-lacZ*-BAC into CBA x C57Bl/6 mouse pronuclei. One transgenic founder mouse was obtained and bred to establish a transgenic mouse line. Integration of two copies of *rBDNF-lacZ*-BAC transgene was estimated by slot-blot hybridization of genomic DNA with [α-^32^P]dCTP-labeled *lacZ*-specific probe.

RNA isolation and analysis of *rBDNF-lacZ mRNA* expression in transgenic mouse tissues with RT-PCR was performed as described (Pruunsild *et al.*, 2007). Quantitative real-time PCR was performed on LightCycler 2.0 (Roche Diagnostics, Mannheim, Germany) using qPCR Core Kit for SYBR^(r)^ Green I No ROX (Eurogentec, Liège, Belgium). qPCR reactions were processed in triplicate and all expression data were normalized to hypoxan-thine phosphoribosyltransferase 1 *(HPRT1)* mRNA levels. For primer sequences see Table 1. In situ hybridization analysis with [α-^35^S]UTP-labeled cRNA probes for *rBDNF-lacZ* and endogenous mouse *BDNF* mRNA was performed as described in Timmusk *et al.* (1993). Kainic acid (KA; 30 mg/kg) or phosphate-buffered saline was administered intraperitoneally to adult *rBDNF-lacZ*-BAC mice weighing 20–25 g. Two kainic acid-treated and two vehicle-treated animals were used for qRT-PCR analysis. Four kainic acid-treated animals and one vehicle-treated animal were used for in situ hybridization analysis. Only animals with induced tonic-clonic seizures were selected for analysis and results are shown for individuals showing highest induction of transgenic and endogenous *BDNF* mRNA. All animal procedures were carried out in compliance with the local ethics committee.

**Table 1:** PCR Primers Used in This Study

BAC modification	
mrBDNF_rpsLneo_F	TGTCTGTCTCTGCTTCCTTCCCACAGTTCCACCAGGTGAGAAGAGTGGGCCTGGTGATGATGGCGGGATCG
rBDNF_rpsLneo_R	ATACAAATAGATAATTTTTGTCTCAATATAATCTATACAACATAAATCCATCAGAAGAACTCGTCAAGAAGG
BDNF_lacZ_300_F	GCCGTCACTTGCTTAGAAACCGTT
BDNF_lacZ_300_R	GAGTACTAACAAGAACGAAGATACT
Genotyping/RT-PCR	
rBDNF_LacZ_F	CCCTGCAGCTGGAGTGGATCAGTAAG
rBDNF_LacZ_R	GAAGATCGCACTCCAGCCAGCTTTCC
mBDNF_F	GTATGTTCGGGCCCTTACTATGGATAGC
mBDNF_R	AAGTTGTGCGCAAATGACTGTTTC
HPRT1_F	CTTTGCTGACCTGCTGGATTAC
HPRT1_R	GTCCTTTTCACCAGCAAGCTTG
Quantitative real-time RT-PCR	
Mouse endogenous mRNAs	
mBDNFq_I_F	TTGAAGCTTTGCGGATATTGCG
mBDNFq_IV_F	GAAATATATAGTAAGAGTCTAGAACCTTG
mBDNFq_VI_F	GCTTTGTGTGGACCCTGAGTTC
mBDNFq_RT_IXcod_R	AAGTTGCCTTGTCCGTGGAC
mBDNFq_cod_F	GGCCCAACGAAGAAAACCAT
mBDNFq_cod_R	AGCATCACCCGGGAAGTGT
HPRT1q_F	CAGTCCCAGCGTCGTGATTA
HPRT1q_R	AGCAAGTCTTTCAGTCCTGTC
Rat BDNF-lacZ mRNAs	
rBDNFq_I_F	AGTCTCCAGGACAGCAAAGC
rBDNFq_IV_F	GAAATATATAGTAAGAGTCTAGAACCTTG
rBDNFq_VI_F	GCTTTGTGTGGACCCTGAGTTC
LacZq_F	CGAAGTGACCAGCGAATACCTGT
LacZq_R1	CAACTGTTTACCTTGTGGAGCGACA
LacZq_R2 (with I_F)	CAAGGCGATTAAGTTGGGTAAC
LacZq_R3 (with IV,VI_F)	GTTTTCCCAGTCACGACGTT
